# Risk of secondary autoimmune diseases with alemtuzumab treatment for multiple sclerosis: a systematic review and meta-analysis

**DOI:** 10.3389/fimmu.2024.1343971

**Published:** 2024-04-16

**Authors:** Jianguo Yang, Yuying Sun, Xinpeng Zhou, Di Zhang, Ziqi Xu, Jiaojiao Cao, Bing Fan

**Affiliations:** ^1^The First School of Clinical Medicine, Shandong University of Traditional Chinese Medicine, Jinan, Shandong, China; ^2^Rheumatology and Immunology Department, Affiliated Hospital of Shandong University of Traditional Chinese Medicine, Jinan, Shandong, China

**Keywords:** secondary autoimmune diseases, alemtuzumab, multiple sclerosis, side effects, meta-analysis

## Abstract

**Background and purpose:**

The objective of this study is to evaluate the risk of secondary autoimmune diseases in multiple sclerosis (MS) patients treated with alemtuzumab (ALZ) through a meta-analysis.

**Methods:**

PubMed, Web of Science, OVID, EMBASE, and Cochrane central register of controlled trials were searched. Information and data were screened and extracted by 2 researchers. The obtained data were analyzed using the R software meta package. Quality assessment was conducted using the Newcastle-Ottawa Scale (NOS). The causes of heterogeneity were analyzed using subgroup analysis and sensitivity analysis. Publication bias was evaluated using funnel plots and Egger’s test.

**Results:**

The search retrieved a total of 3530 papers from the databases. After screening, a total of 37 studies were included in the meta-analysis. The analysis results indicate that the pooled incidence rate of overall secondary autoimmune events (SAEs) in the included studies was 0.2824 [0.2348, 0.3300] (I²=94%, p<0.01). The overall incidence of autoimmune thyroid events (ATE) was 0.2257 [0.1810, 0.2703] (I²=94%, p<0.01). Among them, the rate of serious autoimmune thyroid events (SATE) was 0.0541 [0.0396, 0.0687] (I²=0%, p=0.44). The incidence rates of different thyroid events were as follows: Graves’ disease (GD), 0.2266 [0.1632, 0.2900] (I²=83%, p<0.01); Hashimoto thyroiditis (HT), 0.0844 [0.0000, 0.2262] (I²=81%, p=0.02); Hashimoto thyroiditis with hypothyroidism (HTwH), 0.0499 [0.0058, 0.0940] (I²=37%, p=0.21); fluctuating thyroid dysfunction (FTD), 0.0219 [0.0015, 0.0424] (I²=0%, p=0.40); transient thyroiditis (TT), 0.0178 [0.0062, 0.0295] (I²=0%, p=0.94). The overall incidence of hematological events was 0.0431 [0.0274, 0.0621] (I²=70%, p<0.01). The incidence rates from high to low were as follows: lymphopenia, 0.0367 [0.0000, 0.0776] (I²=81%, p=0.02); Idiopathic thrombocytopenic purpura (ITP), 0.0258 [0.0199, 0.0323] (I²=25%, p=0.15); Hemolytic anemia (HA), 0.0177 [0.0081, 0.0391] (I²=29%, p=0.23); pancytopenia, 0.0136 [0.0000, 0.0314] (I²=0%, p=0.67); Neutropenia, 0.0081 [0.0000, 0.0183] (I²=0%, p=0.42). After excluding thyroid and hematological diseases, the combined incidence of other related SAEs was 0.0061 [0.0014, 0.0109] (I²=50%, p=0.02). The incidence of each disease ranked from highest to lowest as: skin psoriasis (SP), 0.0430 [0.0000, 0.0929] (I²=0%, p=0.57); alopecia areata (AA), 0.0159 [0.0024, 0.0372] (I²=19%, p=0.29); vitiligo, 0.0134 [0.0044, 0.0223] (I²=0%, p=0.81); inflammatory atrichia (IA), 0.0103 [0.0000, 0.0232] (I²=0%, p=0.43); chronic urticaria (CU), 0.0107 [0.0000, 0.0233] (I²=0%, p=0.60); and nephropathy, 0.0051 [0.0000, 0.0263] (I²=62%, p=0.02).

**Conclusion:**

The occurrence of secondary autoimmune diseases in patients with MS treated with ALZ is noteworthy, particularly in the form of thyroid events and hematological events. Clinicians should monitor the overall condition of patients promptly for early management and avoid delayed diagnosis and treatment.

**Systematic review registration:**

inplasy.com/inplasy-2024-4-0048/, identifier INPLASY202440048.

## 1 Introduction

Multiple sclerosis (MS) is an immune-mediated central nervous system demyelinating disease of unknown etiology (1). The majority of MS patients experience reversible episodes of neurological dysfunction and have a remitting-relapsing course (remitting-relapsing MS, RRMS). Currently, two main treatment strategies of MS are commonly employed: escalation therapy or induction of immune reset therapy. Alemtuzumab (ALZ) is one of the cutting-edge therapeutic agents in the MS treatment arsenal. In the escalation therapy strategy, ALZ is commonly considered as a second- or third-line treatment option after inadequate response to conventional disease-modifying therapies (DMTs). Among the induction immune reset strategies, some scholars have advocated the use of ALZ as a first-line medication to induce long-term stability over the course of the disease (2). The mechanism of action of ALZ primarily involves the rapid depletion of white blood cells expressing CD52 through cell lysis, leading to long-term reduction and stabilization of T lymphocytes and inducing a prolonged quiescent state (3). The profound impact of ALZ’s capacity to induce immune reconstitution on the immune system, coupled with the fact that autoimmune diseases tend to arise from lymphocyte depletion, has made secondary autoimmune diseases a relatively common adverse reaction during the use of ALZ (4, 5). Current perspectives suggest that incomplete T-cell repertoire renewal, reduced thymopoiesis, homeostatic proliferation with disparate dynamics of clonal T- and B-cell expansions may be associated with the development of SAEs following the administration of ALZ (6, 7). Furthermore, imbalances among distinct subsets of helper T cells could potentially influence self-reactive T-cell populations, thereby contributing to autoimmune tissue damage (8). The underlying mechanisms behind these conditions, though, currently lack a universally accepted explanation (9, 10).

In summary, ALZ as a significant treatment modality for MS has garnered considerable attention from researchers. However, the occurrence of secondary autoimmune diseases following ALZ infusion should not be overlooked. In light of this, we have conducted a meta-analysis on this topic, aiming to provide comprehensive insights into the incidence of secondary autoimmune diseases related to ALZ and further guide clinical practice.

## 2 Methods

### 2.1 Inclusion and exclusion criteria

The inclusion criteria were as follows: (1) Studies involving individuals diagnosed with multiple sclerosis (MS) according to the McDonald criteria. (2) Studies investigating the therapeutic use of alemtuzumab as the primary intervention. (3) Studies assessing the occurrence of secondary autoimmune diseases in patients receiving ALZ treatment. (4) Clinical studies, including randomized controlled trials, case-control studies, and cohort studies.

The exclusion criteria were as follows: (1) Literature reviews, case reports, commentaries, letters, and conference abstracts. (2) Repetitive publications or studies with duplicated study populations. (3) Studies not published in the English language. (4) Studies not reporting or unable to extract data on secondary autoimmune events.

### 2.2 Search strategy

The search strategy was developed following the PICOS principle, which involved identifying key indexing terms such as “secondary autoimmunity”, “Autoimmunity”, “Autoimmune Diseases”, “Alemtuzumab” and “Multiple Sclerosis”. The search was conducted using MeSH terms and Boolean operators. The detailed search strategy can be found in the **Supplementary Materials**. Renowned databases, including PubMed, Web of Science, OVID, EMBASE, and the Cochrane Library, were comprehensively searched to obtain relevant literature. The search results were then imported into EndNote X9.1 for efficient literature management. Two researchers conducted the initial screening of the retrieved articles based on reference relevance. In the event of any disagreement during the article selection process, a third author was consulted for resolution. The search was conducted up to May 2023.

### 2.3 Data extraction

Two investigators conducted a comprehensive review and data extraction for the eligible studies meeting the inclusion criteria. A table was constructed, encompassing essential details such as the primary author, publication year, diagnostic criteria, age, gender, follow-up duration, country or region, number of patients included who met the criteria, the final incidence of Secondary autoimmune events (SAEs) in patients, and the occurrence of each specific autoimmune disease. In the case of any discrepancies, they were resolved through discussion and consensus. If consensus could not be reached between the two researchers, the matter was referred to a third investigator for final judgment.

### 2.4 Quality assessment

The quality assessment of the included studies was conducted using the Newcastle-Ottawa Scale (NOS), which can be accessed at http://www.ohri.ca/programs/clinical_epidemiology/oxford.asp. Studies with NOS scores exceeding 5 points were deemed eligible for inclusion in the meta-analysis.

### 2.5 Data analysis

The meta-analysis was conducted using the “meta/metafor” package in R 4.2.2 software (11). Firstly, the original rates were transformed using logarithmic, logit, arcsine, and Freeman-Tukey double arcsine transformations. The Shapiro-Wilk normality test was employed to assess the normality of each dataset, and the appropriate transformation method was chosen based on the distribution. The overall incidence rate of secondary autoimmune diseases in MS patients treated with ALZ was then calculated, along with its 95% confidence interval (CI). The heterogeneity among the included studies was evaluated using the Cochrane Q test and I² statistic. If the Cochrane Q test yielded a p-value of ≤0.05 or I² was ≥50%, significant heterogeneity was deemed present. In such cases, sensitivity analysis was conducted by omitting individual studies sequentially to assess the stability of the combined results. Studies that had an abnormal influence on the analysis results were excluded on a discretionary basis to observe if heterogeneity was eliminated.

Subgroup analysis was considered to observe whether heterogeneity was reduced among different subgroups. If the heterogeneity remained after these steps, a random-effects model was used to calculate the combined rate and its 95% CI, with a careful analysis of the source of heterogeneity. In the absence of significant heterogeneity, a fixed-effects model was used to combine the overall rates. Finally, a funnel plot and Egger’s test were used to assess publication bias. In the presence of clear outliers, the possibility of excluding such studies may be considered after a meticulous analysis of potential sources of bias.

The conduct of this meta-analysis was consistent with the guidelines in the Preferred Reporting Items for Systematic Reviews and Meta-analysis (PRISMA) statement (**Supplementary Materials**).

## 3 Results

### 3.1 Characteristics and quality of the included studies

**Figure 1:**
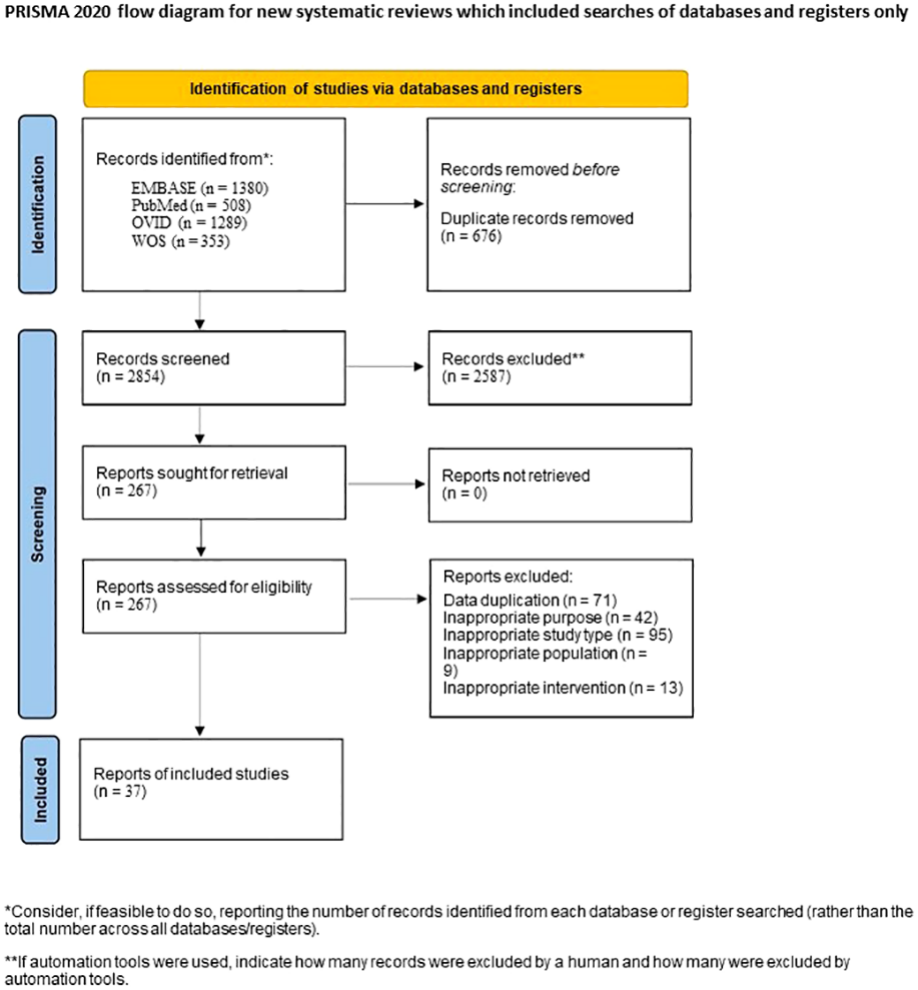
Flow chart of study selection process.

Through the database search, a total of 3530 articles were initially identified. After the preliminary screening and subsequent full-text assessment, a final selection of 37 studies was included in the analysis (**Table 1**, **Figure 1**) (12–48). All of the included studies were written in English, with 26 studies were published in 2020 or later. These studies collectively involved 4171 patients, among whom 1151 patients developed SAEs. The quality assessment using the NOS indicated scores of ≥5 for all studies, further details of which are presented in the **Supplementary Materials**.

**Table 1:** Characteristics and quality assessment of eligible studies in meta-analysis.

name	study	countrty	population with ALZ	Autoimmune events	Types of Autoimmune events	Quality score
Alroughani 2023 (12)	prospective	Kuwait	73	12	Hyperthyroidism, hypothyroidism, SH	6
Eichau 2023 (13)	retrospective	Spain	123	27	Hyperthyroidism, hypothyroidism, ITP, Vitiligo, AA, CU	7
Kazakou 2023 (14)	prospective	Greece	35	17	GD, HT, HTwH, HwPT	6
Sandgren 2023 (15)	prospective	Sweden	124	55	ATE, GD, TT, ITP, neutropenia, HA	6
Signoriello 2023 (16)	retrospective	Italy	150	22	ATE	7
Pfeuffer 2023 (17)	retrospective	Germany	170	52	ATE, GD, ITP, neutropenia, Vitiligo, Autoimmune hepatitis, Idiopathic Castleman’s disease	6
Bónitto 2022 (18)	retrospective	Colombia	23	2	ATE, Papillary thyroid carcinoma	6
Lopez Ruiz 2022 (19)	retrospective	Spain	133	8	Vitiligo, AA, CU, IA	5
Manso 2022 (20)	retrospective	Italy	57	22	GD, HTwH, HwPT, SH, silent thyroiditis	7
Palmeri 2022 (21)	prospective	Italy	31	13	ATE, ITP, SP	6
Rauma 2022 (22)	retrospective	Finland	121	37	ATE, Serious autoimmune thyroid event, ITP, lymphopenia, Acute acalculous cholecystitis, Malignant disease	7
Rodríguez de Vera Gómez 2022 (23)	retrospective	spain	121	41	ATE, GD, Graves’ orbitopathy	7
Russo 2022 (24)	retrospective	Italy	322	34	ATE	6
Alping 2021 (25)	retrospective	Sweden	132	33	ATE	7
Bass 2021 (26)	prospective	multicenter	811	386	ATE, SATE, ITP, Nephropathy	7
Bose 2021 (27)	retrospective	Canada	46	25	ATE, ITP, HA	8
Brecl Jakob 2021 (28)	retrospective	Slovenia and Croatia	71	27	ATE, hyperthyroidism, hypothyroidism, FTD, ITP, Nephropathy, follicular adenoma of the thyroid gland	7
Häußler 2021 (29)	prospective	Germany	21	6	ATE, ITP, AA	8
Herman 2021 (30)	retrospective	US	60	–	ATE, ITP	7
Theodorsdottir 2021 (31)	prospective	Denmark	209	31	ATE, ITP, HA, Nephropathy, Rheumatoid arthritis, Thrombocytosis	7
Walo-Delgado 2021 (32)	prospective	Spain	57	22	ATE, ITP	6
Boffa 2020 (33)	prospective	Italy	32	14	ATE, ITP, SP, Nephropathy, Myositis, Asthma	7
di Ioia 2020 (34)	prospective	Italy	35	15	ATE, ITP, HA, Pancytopenia, Nephropathy	7
Rodríguez de Castro 2020 (35)	retrospective	Spain	23	11	GD, hyperthyroidism, hypothyroidism, ITP	5
Sovetkina 2020 (36)	retrospective	UK	126	33	ATE, GD, TT, hyperthyroidism, hypothyroidism, FTD	7
Yap 2020 (37)	retrospective	Ireland	52	16	ATE, GD, Graves’ orbitopathy	7
Alcalá 2019 (38)	prospective	Spain	28	5	ATE, Pancytopenia	7
Frau 2019 (39)	prospective	Italy	90	13	ATE, ITP	6
Kim 2019 (40)	retrospective	South Korea	19	0	Malignant disease	5
Ruck 2019 (8)	prospective	Germany	106	31	ATE, ITP, Vitiligo	7
Kocsik 2018 (41)	retrospective	US	29	6	ATE, ITP, Nephropathy, SLE-like syndrome of cutaneous vasculitis/inflammatory arthritis	6
Muller 2018 (42)	retrospective	UK	45	–	GD, HwPT, chronic ATE, TPOAb+ subacute thyroiditis, TPOAb-/TRAb- hypothyroidism	7
Pariani 2018 (43)	retrospective	UK	248	–	ATE, GD, HT (HT), TT, hyperthyroidism, hypothyroidism, HwPT	6
Prosperini 2018 (44)	retrospective	Italy	40	11	ATE, ITP, Exfoliative dermatitis, Impetigo	7
Wang 2018 (38)	prospective	China	292	74	ATE, ITP, lymphopenia	6
Willis 2016 (45)	prospective	UK	100	47	ATE, ITP, HA, Pancytopenia, Vitiligo, IA, transient infusion related thrombocytopaenia, neutropaenia, autoimmune alveolitis, autoimmune hepatitis, type II diabetes mellitus, anti-phospholipid syndrome	7
Le Page 2015 (46)	prospective	France	16	3	ATE, GD, ITP	7

AA, alopecia areata; ATE, autoimmune thyroid events; CU, chronic urticaria; FTD, fluctuating thyroid dysfunction; GD, Graves’ disease; HA, Hemolytic anemia; HT, Hashimoto thyroiditis; HTwH, Hashimoto thyroiditis with hypothyroidism; HwPT, hypothyroidism with positive TRAb; IA, inflammatory atrichia; ITP, Idiopathic thrombocytopenic purpura; MS, multiple sclerosis; SATE, serious autoimmune thyroid events; SH, subclinical hypothyroidism; SP, skin psoriasis; TT, transient thyroiditis.

### 3.2 Overall incidence of secondary autoimmune events

**Figure 2:**
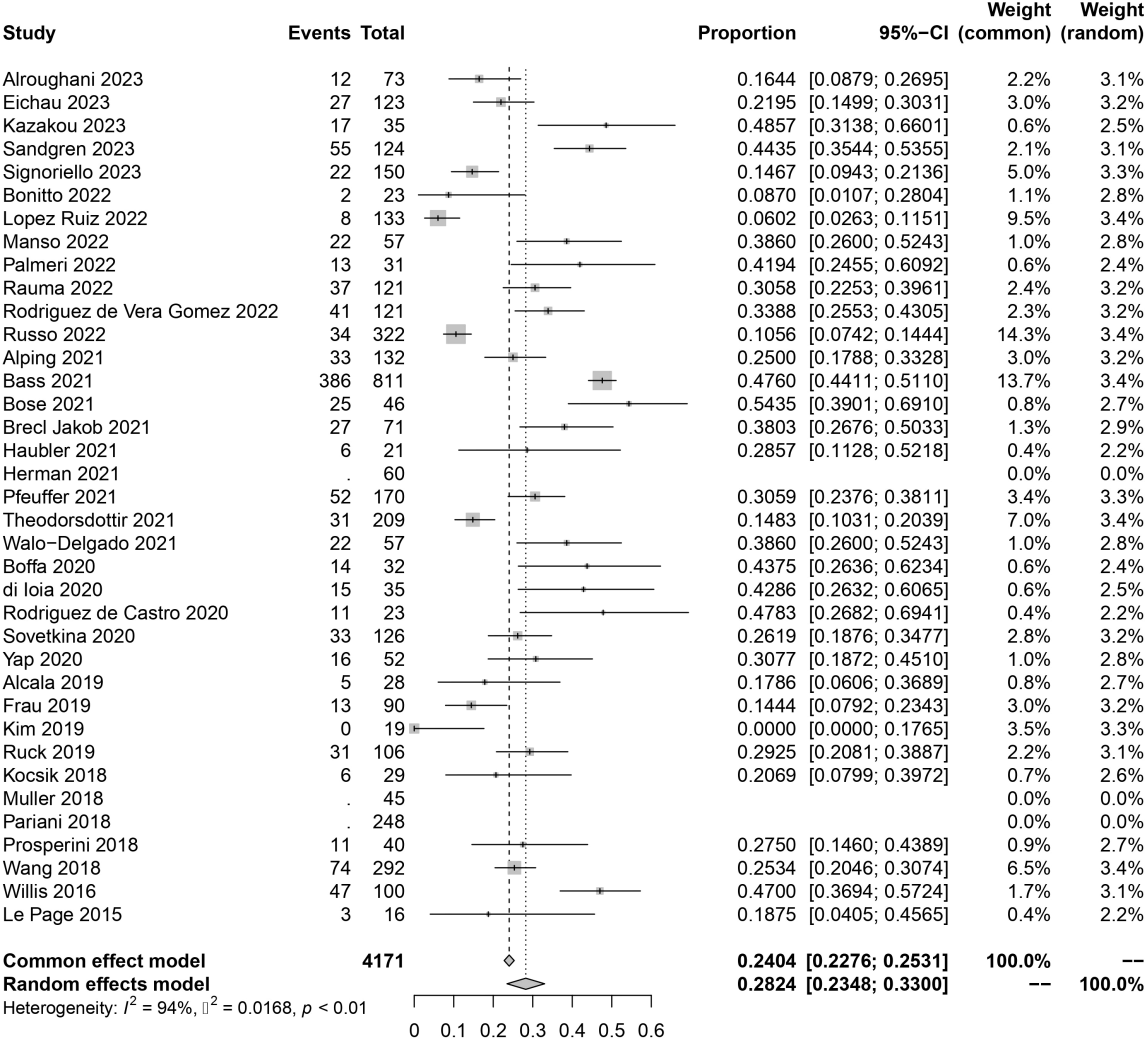
Forest plot of autoimmune events.

The original data of SAEs demonstrated a normal distribution, allowing for the use of raw data in the meta-analysis. The overall rate obtained was 0.2824 [0.2348, 0.3300] (I²=94%, p<0.01) (**Figure 2**), indicating significant heterogeneity among the included studies according to the Cochrane Q test and I² test. No evident publication bias was detected based on the funnel plot and Egger’s test (t = 1.52, p=0.1394) (**Supplementary Materials**). Sensitivity analysis through sequential omission did not identify any studies with abnormal influences on the results. Therefore, a random-effects model was employed to describe the combined results. Subgroup analysis will be performed in subsequent sections.

### 3.3 Incidence of autoimmune thyroid events

**Figure 3:**
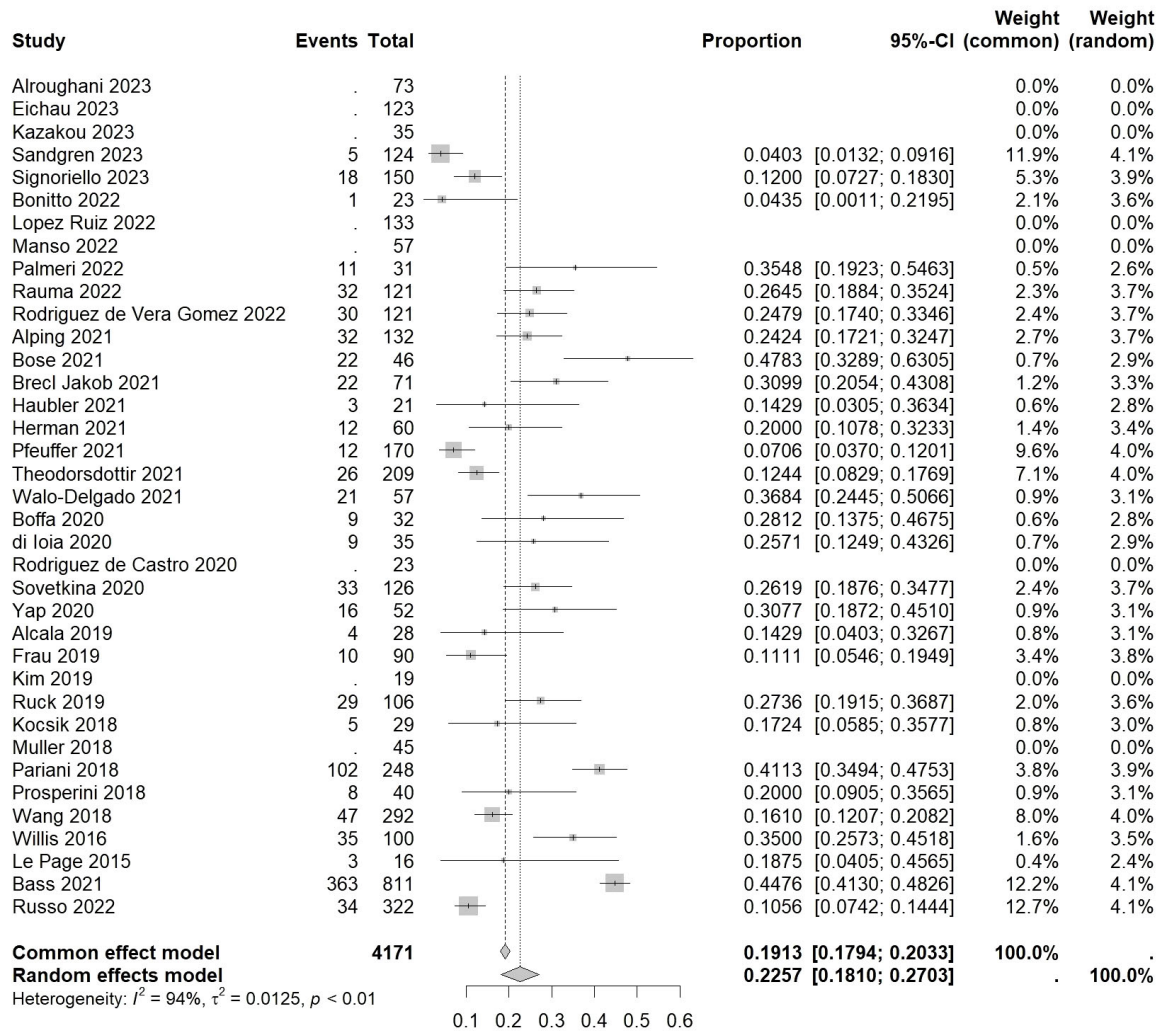
Forest plot of autoimmune thyroid events.

For autoimmune thyroid events, the overall incidence rate of autoimmune thyroid events (ATE) was found to be 0.2257 [0.1810, 0.2703] (I²=94%, p<0.01) (**Figure 3**). As for serious autoimmune thyroid events (SATE), the rate was 0.0541 [0.0396, 0.0687] (I²=0%, p=0.44) (**Table 2**).

**Table 2:** Meta-analysis of different diseases.

	Rate	LCL	UCL	I²	p
Overall incidence of secondary autoimmune events					
overall rate	0.2824	0.2348	0.3300	94%,	<0.01
Incidence of thyroid-associated autoimmune events					
ATE	0.2257	0.1810	0.2703	94%	<0.01
SATE	0.0541	0.0396	0.0687	0%	0.44
GD	0.2266	0.1623	0.2900	83%	<0.01
HT	0.0844	0.0000	0.2262	81%	0.02
HTwH	0.0499	0.0058	0.0940	37%	0.21
FTD	0.0219	0.0015	0.0424	0%	0.40
TT	0.0178	0.0062	0.0295	0%	0.94
hyperthyroidism	0.0586	0.0158	0.1015	81%	<0.01
hypothyroidism	0.0591	0.0197	0.0985	81%	<0.01
SH	0.0569	0.0174	0.0964	47%	0.17
HwPT	0.0520	0.0336	0.0806	0%	0.57
Incidence of hematologic related autoimmune events					
overall rate of hematological disorders	0.0431	0.0274	0.0621	70%	<0.01
lymphopenia	0.0367	0.0000	0.0776	81%	0.02
ITP	0.0258	0.0199	0.0323	25%	0.15
HA	0.0177	0.0081	0.0391	29%	0.23
pancytopenia	0.0136	0.0000	0.0314	0%	0.67
Neutropenia	0.0081	0.0000	0.0183	0%	0.42
Incidence of other autoimmune disease events					
overall rate of other SAEs	0.0061	0.0014	0.0109	50%	0.02
SP	0.0430	0.0000	0.0929	0%	0.57
AA	0.0159	0.0024	0.0372	19%	0.29
vitiligo	0.0134	0.0044	0.0223	0%	0.81
IA	0.0103	0.0000	0.0232	0%	0.43
CU	0.0107	0.0000	0.0233	0%	0.60
nephropathy	0.0051	0.0000	0.0263	62%	0.02

AA, alopecia areata; ATE, autoimmune thyroid events; CU, chronic urticaria; FTD, fluctuating thyroid dysfunction; GD, Graves’ disease; HA, Hemolytic anemia; HT, Hashimoto thyroiditis; HTwH, Hashimoto thyroiditis with hypothyroidism; HwPT, hypothyroidism with positive TRAb; IA, inflammatory atrichia; ITP, Idiopathic thrombocytopenic purpura; SATE, serious autoimmune thyroid events; SH, subclinical hypothyroidism; SP, skin psoriasis; TT, transient thyroiditis.

Among specific disease subtypes, the highest incidence rate was observed in Graves’ disease (GD), with a rate of 0.2266 [0.1632, 0.2900] (I²=83%, p<0.01). This was followed by Hashimoto thyroiditis (HT) with a rate of 0.0844 [0.0000, 0.2262] (I²=81%, p=0.02). Among patients undergoing Hashimoto thyroiditis with hypothyroidism (HTwH), the incidence rate was 0.0499 [0.0058, 0.0940] (I²=37%, p=0.21). Finally, fluctuating thyroid dysfunction (FTD) had an incidence rate of 0.0219 [0.0015, 0.0424] (I²=0%, p=0.40), and transient thyroiditis (TT) had a rate of 0.0178 [0.0062, 0.0295] (I²=0%, p=0.94).

When considering thyroid function classification alone, the overall incidence rate of hyperthyroidism was 0.0586 [0.0158, 0.1015] (I²=81%, p<0.01), while hypothyroidism had an incidence rate of 0.0591 [0.0197, 0.0985] (I²=81%, p<0.01), and subclinical hypothyroidism (SH) had a rate of 0.0569 [0.0174, 0.0964] (I²=47%, p=0.17). Additional subtypes, such as hypothyroidism with positive TRAb (HwPT), had an incidence rate of 0.0520 [0.0336, 0.0806] (I²=0%, p=0.57).

### 3.4 Incidence of autoimmune hematologic events

**Figure 4:**
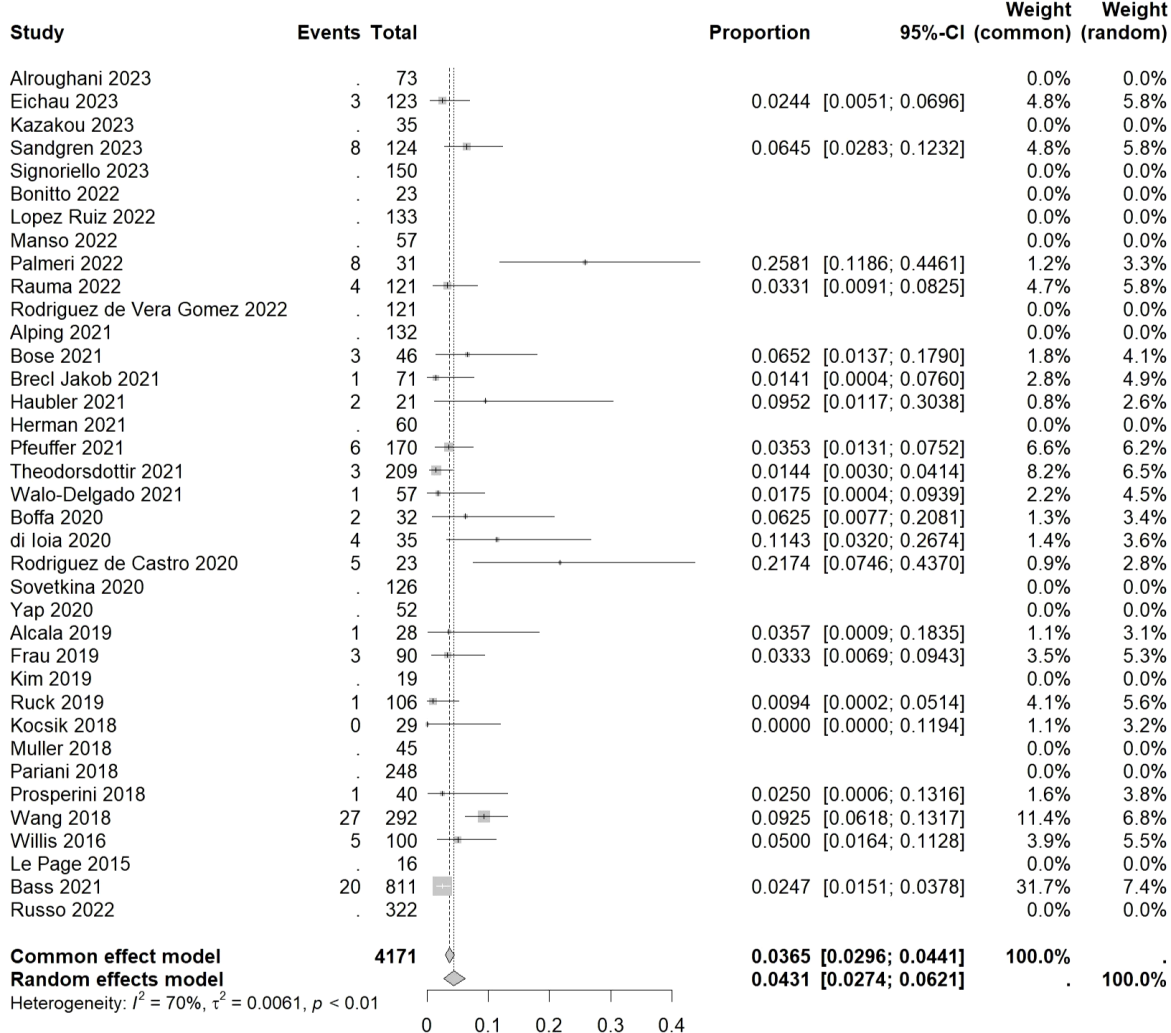
Forest plot of autoimmune hematologic events.

The overall incidence rate of autoimmune hematologic events was found to be 0.0431 [0.0274, 0.0621] (I²=70%, p<0.01) (**Figure 4**).

Among hematological disorders, lymphopenia had the highest incidence rate of 0.0367 [0.0000, 0.0776] (I²=81%, p=0.02), followed by idiopathic thrombocytopenic purpura (ITP), hemolytic anemia (HA), and pancytopenia, with incidence rates of 0.0258 [0.0199, 0.0323] (I²=25%, p=0.15), 0.0177 [0.0081, 0.0391] (I²=29%, p=0.23), and 0.0136 [0.0000, 0.0314] (I²=0%, p=0.67), respectively. The lowest incidence rate was observed in neutropenia at 0.0081 [0.0000, 0.0183] (I²=0%, p=0.42).

### 3.5 Incidence of other autoimmune disease events

Excluding thyroid and hematological disorders, the overall incidence rate of other SAEs was found to be 0.0061 [0.0014, 0.0109] (I²=50%, p=0.02).

Among these, the highest incidence rate was observed in skin psoriasis (SP) at 0.0430 [0.0000, 0.0929] (I²=0%, p=0.57). This was followed by alopecia areata (AA), vitiligo, inflammatory atrichia (IA), chronic urticaria (CU), and nephropathy. The summarized incidence rates for each of these conditions are as follows: AA had a rate of 0.0159 [0.0024, 0.0372] (I²=19%, p=0.29), vitiligo had a rate of 0.0134 [0.0044, 0.0223] (I²=0%, p=0.81), IA had a rate of 0.0103 [0.0000, 0.0232] (I²=0%, p=0.43), CU had a rate of 0.0107 [0.0000, 0.0233] (I²=0%, p=0.60), and nephropathy had a rate of 0.0051 [0.0000, 0.0263] (I²=62%, p=0.02).

### 3.6 Subgroup analysis

We conducted subgroup analysis of overall SAEs based on publication year and geographical region. The results are as follows: for studies published before 2020, the overall incidence rate of SAEs was 0.2717 [0.1988, 0.3446] (I²=86%, p<0.01), while for studies published in 2021 and onwards, the rate was 0.2892 [0.2253, 0.3532] (I²=96%, p<0.01). In the subgroup analysis based on geographic region, the incidence rate for the Asian subgroup was 0.1403 [0.0000, 0.2879] (I²=94%, p<0.01), for the American subgroup it was 0.1324 [0.0417, 0.2232] (I²=37%, p=0.21), and for the European subgroup it was 0.2987 [0.2498, 0.3475] (I²=90%, p<0.01).

Regarding thyroid events and hematological system events, we did not observe a significant reduction in heterogeneity through subgroup analysis based on time and region, and therefore, we will not provide a detailed description of these analysis.

### 3.7 Sensitivity analysis

Our research findings indicate that for the meta-analysis of various diseases, the sequential omission of any individual study did not significantly influence the results. Consequently, we conclude that the observed high heterogeneity in the analysis results is not attributable to the impact of individual studies on the overall data. Heterogeneity has other origins. Further analysis related to this will be elaborated in the discussion section.

## 4 Discussion

In this systematic review and meta-analysis, our primary focus has been on the occurrence rates of autoimmune events in patients with MS treated with ALZ. The results indicate that approximately 28% of MS patients experienced autoimmune events following ALZ treatment. Among these events, thyroid-related events demonstrated the highest incidence, with the descending order of incidence rates as follows: GD, HT, HTwH, FTD, and TT. In terms of thyroid function, the incidence rates of hyperthyroidism, hypothyroidism, SH, and HwPT were relatively similar. The overall incidence rate of hematologic events was lower than that of thyroid events, with lymphopenia, ITP, HA, pancytopenia, and Neutropenia ranked from highest to lowest. The incidence rates of other autoimmune events were comparatively lower, specifically including SP, AA, vitiligo, IA, CU, and nephropathy.

Our study indicates that the results of meta-analysis pooling data from various diseases exhibit a certain degree of heterogeneity, and despite subgroup analysis and sensitivity analysis, the specific sources of this heterogeneity remain unclear. Therefore, further analysis is required to investigate the heterogeneity and bias in the study population.

From the perspective of researchers, firstly, variations in the definition of secondary autoimmune diseases across different regions and studies result in differences among the patient populations included. Additionally, some researchers may not include cases of milder or transient autoimmune diseases that are difficult to diagnose in their observations. Moreover, inherent diagnostic suspicion bias among researchers leads to subjective biases and tendencies in the disease diagnosis process. These sources of bias cannot be overlooked in this study. From the standpoint of interventions and patients, factors such as the use of DMTs before using ALZ, washout period duration, concurrent use of corticosteroids to alleviate infusion reactions, etc., may influence the efficacy of immune reconstitution and the occurrence of adverse events related to ALZ (49, 50). Furthermore, differences in treatment regimens and overall treatment conditions across different regions contribute to inherent population variances among patients in different countries. Considering the high cost of ALZ, there is also a certain heterogeneity among populations in different regions based on their varying levels of affluence. Additionally, our included studies primarily focused on the European region, hence the results obtained may exhibit a certain degree of geographical bias, leaning towards the occurrence rates of SAEs following the use of ALZ within the European MS population. This holds particular clinical significance for rheumatologists practicing in Europe.

For autoimmune thyroid events, different researchers present varying perspectives on describing thyroid events. Some researchers classify different thyroid events based on antibodies (43), while others categorize them according to the type of thyroid disease (14). Some researchers simply categorize autoimmune thyroid events based on hyperthyroidism and hypothyroidism (13, 28). Therefore, the overall incidence rate of autoimmune thyroid events obtained can only partially reflect the actual incidence rate. For clinical researchers, clearly defined thyroid function abnormalities are generally not misdiagnosed, providing a certain degree of credibility to the overall incidence rates of thyroid events. However, for more specific subtypes of thyroid events, there may be overlaps, statistical biases, and inherent biases in the research process. These factors significantly influence the biases relevant to this area.

In contrast to thyroid autoimmune events, the incidence rates and heterogeneity derived from meta-analysis of hematological and other autoimmune diseases are relatively low, indicating that these results can effectively reflect the actual occurrence rates of the related diseases. This may be attributed to the fact that hematological diseases and other autoimmune diseases are mainly diagnosed through routine blood tests and symptoms, leading to relatively clear diagnoses. It should be noted, however, that the number of studies included in meta-analysis of hematological and other autoimmune diseases is limited, which could also contribute to the lower heterogeneity observed. Apart from events related to the thyroid and hematological systems, several studies have reported other SAEs following the administration of ALZ, such as sarcoidosis, autoimmune hepatitis, asthma, idiopathic Castleman’s disease, among others (17, 33, 51). These relatively rare complications should also be acknowledged by clinicians.

In recent years, meta-analysis examining adverse reactions to ALZ therapy in MS have primarily focused on thyroid events and infection-related events, both of which are associated with ALZ’s immunoclearance therapeutic mechanism. In 2020, Scappaticcio et al. provided an overview of the overall incidence rate of thyroid events (52). Their study reported an incidence rate of autoimmune thyroid events as 0.33 [0.24-0.43], which differs significantly from our study’s findings. It is important to note that their study presented the proportion of different thyroid diseases contributing to thyroid events in patients with MS, rather than calculating the incidence rate among MS patients undergoing ALZ therapy. This study identified GD as the most common thyroid event, followed by Hashimoto’s thyroiditis, consistent with our findings. Furthermore, Buonomo et al. (2021) investigated the incidence rate of infection events in the target patient population, reporting an incidence rate of 24% (95% CI: 12%-43%) (I²= 97.2%, p < 0.001) (53). Both the studies by Scappaticcio et al. (2020) and Buonomo et al. (2021) exhibited notable heterogeneity in their pooled meta-analysis results, consistent with our study. Apart from these studies, no other meta-analysis on adverse reactions following ALZ therapy in MS were identified in our search.

In terms of the strengths and limitations of this study, the majority of studies included in this meta-analysis were conducted within the past 5 years, which is of significant clinical importance for current clinical practice. Based on the overall incidence rate derived from our meta-analysis, a considerable proportion of MS patients receiving ALZ therapy experienced autoimmune-related diseases. These finding aids clinical practitioners in assessing the benefits and risks for patients, with adverse reactions being a crucial component of this assessment process. Equally important, this study also contributes to predicting patient prognosis, as well as providing MS patients with certain psychological expectations prior to using ALZ, thereby reducing potential conflicts between healthcare providers and patients.

Nevertheless, the included studies exhibit various significant biases that cannot be overlooked, with certain endpoints showing high heterogeneity, necessitating cautious consideration when applying some of our study results to clinical practice. Additionally, although our study findings suggest lower heterogeneity in the blood system compared to other autoimmune events, the incidence rate is also lower. Based on the available evidence, we cannot rule out the impact of baseline occurrence rates of these two types of adverse events on the study results, nor can we definitively establish a causal relationship between these adverse events and the use of ALZ. Therefore, careful interpretation of the clinical significance of this portion of the incidence rates is warranted. For diseases with lower incidence rates and limited study numbers, conducting detailed large-sample controlled trials for comparative analysis is necessary to derive more reliable conclusions.

Healthcare decision-makers should be familiar with and consider the potential adverse reactions associated with ALZ. A certain level of psychological preparedness in this regard is crucial. Before initiating ALZ therapy, the pros and cons of this “immunodepletion” therapy must be carefully evaluated for the patient (54). In terms of clinical research, the current clinical studies on ALZ-related adverse reactions are incomplete. In addition to the CARE-MS trials, more large-scale, multicenter clinical trials should be conducted to obtain additional frontline clinical data (26). Future clinical research is also expected to incorporate more refined and comprehensive stratifications to derive more reliable and trustworthy conclusions. Furthermore, we hope that cellular and molecular research will further advance the understanding of the specific mechanisms and pathways underlying the development of secondary autoimmune diseases post-MS treatment with ALZ. This will provide supplementary guidance for clinical practice and clinical trial research.

## 5 Conclusion

In general, MS patients treated with ALZ exhibit a certain incidence of autoimmune events, including thyroid autoimmune events such as GD, HT, HTwH, FTD, and TT. Autoimmune events involving the hematologic system include lymphopenia, ITP, HA, pancytopenia, and Neutropenia. Other autoimmune events comprise SP, AA, vitiligo, IA, CU, and nephropathy. Further research in this area necessitates larger sample sizes, well-defined multicenter clinical trials to support meta-analysis, and the inclusion of healthy control populations to elucidate the impact of ALZ on the incidence of low-prevalence diseases.

## Data availability statement

The original contributions presented in the study are included in the article/**Supplementary Material**. Further inquiries can be directed to the corresponding author.

## Author contributions

JY: Writing – review & editing, Writing – original draft, Visualization, Software, Methodology, Formal analysis, Data curation, Conceptualization. YS: Writing – original draft, Visualization, Validation, Supervision, Investigation, Formal analysis, Conceptualization. XZ: Writing – original draft, Software, Project administration, Methodology, Formal analysis, Data curation, Conceptualization. DZ: Writing – original draft, Investigation, Data curation, Conceptualization. ZX: Writing – review & editing, Project administration, Methodology, Investigation, Data curation. JC: Writing – original draft, Visualization, Validation, Data curation, Conceptualization. BF: Writing – review & editing, Supervision, Software, Resources, Funding acquisition, Conceptualization.
