# Supplementary figures and images for: Risk of secondary autoimmune diseases with alemtuzumab treatment for multiple sclerosis: a systematic review and meta-analysis
Source: Front Immunol. 2024 Apr 16;15:1343971. doi: 10.3389/fimmu.2024.1343971 (PMC11058189; doi:10.3389/fimmu.2024.1343971)

Standardised treatment effect (z-score)

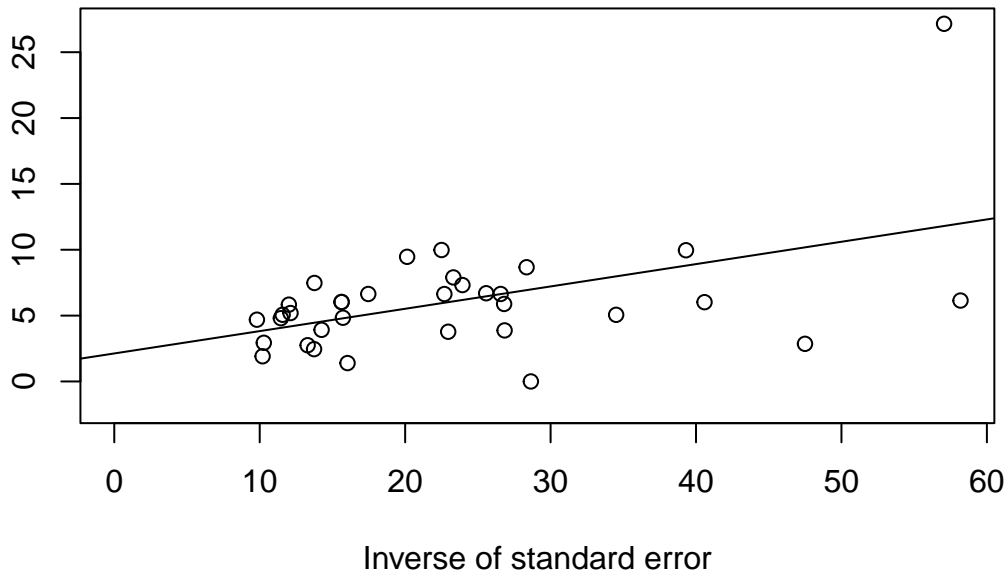

Supplement: Supplementary file 1 [file DataSheet_1.pdf]

Standard Error

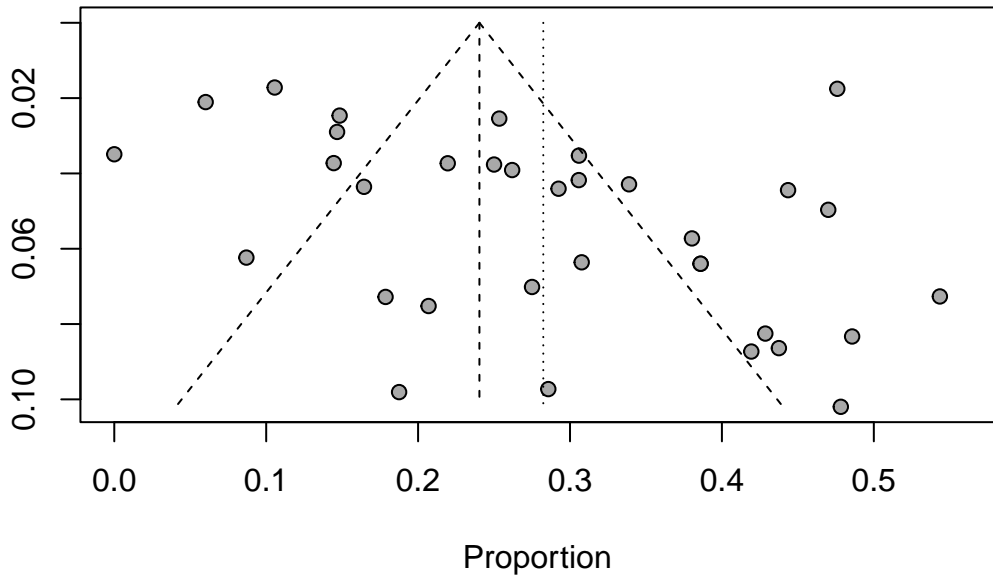

Supplement: Supplementary file 2 [file DataSheet_2.pdf]
