# Supplementary material for: Risk of secondary autoimmune diseases with alemtuzumab treatment for multiple sclerosis: a systematic review and meta-analysis
Source: Front Immunol. 2024 Apr 16;15:1343971. doi: 10.3389/fimmu.2024.1343971 (PMC11058189; doi:10.3389/fimmu.2024.1343971)
Supplement: Supplementary file 3 [file Table_1.docx]

| name | selection | | | | | | | | | | | | | Comparability | | outcome | | | | | | | | | | score |
| --- | --- | --- | --- | --- | --- | --- | --- | --- | --- | --- | --- | --- | --- | --- | --- | --- | --- | --- | --- | --- | --- | --- | --- | --- | --- | --- |
|  | 1) | | | | 2) | | | 3) | | | | 4) | | 5) | | 6) | | | | 7) | | 8) | | | |  |
|  | 1a) | 1b) | 1c) | 1d) | 2a) | 2b) | 2c) | 3a) | 3b) | 3c) | 3d) | 4a) | 4b) | 5a) | 5b) | 6a) | 6b) | 6c) | 6d) | 7a) | 7b) | 8a) | 8b) | 8c) | 8d) |  |
| Signoriello  2023 | ⚫ |  |  |  |  |  |  | ⚫ |  |  |  | ⚫ |  | ⚫ |  |  | ⚫ |  |  | ⚫ |  | ⚫ |  |  |  | 7 |
| Sandgren  2023 |  | ⚫ |  |  |  |  |  |  | ⚫ |  |  |  | ⚫ | ⚫ |  | ⚫ |  |  |  | ⚫ |  | ⚫ |  |  |  | 6 |
| Pfeuffer  2023 |  | ⚫ |  |  |  |  |  | ⚫ |  |  |  | ⚫ |  | ⚫ |  |  |  |  | ⚫ | ⚫ |  | ⚫ |  |  |  | 6 |
| Kazakou  2023 |  | ⚫ |  |  |  |  |  |  | ⚫ |  |  |  | ⚫ | ⚫ |  |  | ⚫ |  |  | ⚫ |  | ⚫ |  |  |  | 6 |
| Eichau  2023 | ⚫ |  |  |  |  |  |  | ⚫ |  |  |  | ⚫ |  | ⚫ |  |  | ⚫ |  |  | ⚫ |  | ⚫ |  |  |  | 7 |
| Alroughani  2023 |  | ⚫ |  |  |  |  |  |  | ⚫ |  |  | ⚫ |  | ⚫ |  |  | ⚫ |  |  | ⚫ |  |  |  |  | ⚫ | 6 |
| Russo  2022 | ⚫ |  |  |  |  |  |  | ⚫ |  |  |  | ⚫ |  | ⚫ |  |  | ⚫ |  |  |  | ⚫ | ⚫ |  |  |  | 6 |
| Rauma  2022 |  | ⚫ |  |  |  |  |  |  | ⚫ |  |  | ⚫ |  | ⚫ |  |  | ⚫ |  |  | ⚫ |  | ⚫ |  |  |  | 7 |
| Rodríguez de  Vera Gómez  2022 | ⚫ |  |  |  |  |  |  |  | ⚫ |  |  | ⚫ |  | ⚫ |  |  | ⚫ |  |  | ⚫ |  |  | ⚫ |  |  | 7 |
| Palmeri  2022 | ⚫ |  |  |  |  |  |  | ⚫ |  |  |  |  | ⚫ | ⚫ |  |  | ⚫ |  |  | ⚫ |  |  | ⚫ |  |  | 6 |
| Manso  2022 |  | ⚫ |  |  |  |  |  | ⚫ |  |  |  | ⚫ |  | ⚫ |  |  | ⚫ |  |  | ⚫ |  |  | ⚫ |  |  | 7 |
| L ́opez Ruiz  2022 |  | ⚫ |  |  |  |  |  | ⚫ |  |  |  | ⚫ |  | ⚫ |  |  | ⚫ |  |  |  | ⚫ |  |  |  | ⚫ | 5 |
| B ́onitto  2022 | ⚫ |  |  |  |  |  |  | ⚫ |  |  |  | ⚫ |  | ⚫ |  |  | ⚫ |  |  |  | ⚫ | ⚫ |  |  |  | 6 |
| Theodorsdottir  2021 | ⚫ |  |  |  |  |  |  |  | ⚫ |  |  | ⚫ |  | ⚫ |  |  | ⚫ |  |  | ⚫ |  |  | ⚫ |  |  | 7 |
| Delgado  2021 | ⚫ |  |  |  |  |  |  |  | ⚫ |  |  |  | ⚫ | ⚫ |  |  | ⚫ |  |  | ⚫ |  |  | ⚫ |  |  | 6 |
| Herman  2021 |  | ⚫ |  |  |  |  |  | ⚫ |  |  |  | ⚫ |  | ⚫ |  |  | ⚫ |  |  | ⚫ |  | ⚫ |  |  |  | 7 |
| Häußler  2021 |  | ⚫ |  |  | ⚫ |  |  |  | ⚫ |  |  | ⚫ |  | ⚫ |  |  | ⚫ |  |  | ⚫ |  | ⚫ |  |  |  | 8 |
| Jakob  2021 | ⚫ |  |  |  |  |  |  | ⚫ |  |  |  | ⚫ |  | ⚫ |  |  | ⚫ |  |  | ⚫ |  |  | ⚫ |  |  | 7 |
| Bose  2021 |  | ⚫ |  |  | ⚫ |  |  | ⚫ |  |  |  | ⚫ |  | ⚫ |  |  | ⚫ |  |  | ⚫ |  | ⚫ |  |  |  | 8 |
| Bass  2021 |  | ⚫ |  |  |  |  |  | ⚫ |  |  |  | ⚫ |  | ⚫ |  |  | ⚫ |  |  | ⚫ |  |  | ⚫ |  |  | 7 |
| Alping  2021 |  | ⚫ |  |  | ⚫ |  |  | ⚫ |  |  |  |  | ⚫ | ⚫ |  |  | ⚫ |  |  | ⚫ |  | ⚫ |  |  |  | 7 |
| Yap  2020 |  | ⚫ |  |  |  |  |  | ⚫ |  |  |  | ⚫ |  | ⚫ |  |  | ⚫ |  |  | ⚫ |  |  | ⚫ |  |  | 7 |
| Sovetkina  2020 |  | ⚫ |  |  | ⚫ |  |  | ⚫ |  |  |  |  | ⚫ | ⚫ |  |  | ⚫ |  |  | ⚫ |  |  | ⚫ |  |  | 7 |
| Rodríguez de  Castro  2020 |  | ⚫ |  |  |  |  |  | ⚫ |  |  |  |  | ⚫ | ⚫ |  |  | ⚫ |  |  |  | ⚫ | ⚫ |  |  |  | 5 |
| Di Ioia  2020 |  | ⚫ |  |  |  |  |  |  | ⚫ |  |  | ⚫ |  | ⚫ |  |  | ⚫ |  |  | ⚫ |  | ⚫ |  |  |  | 7 |
| Boffa  2020 |  | ⚫ |  |  | ⚫ |  |  |  | ⚫ |  |  |  | ⚫ | ⚫ |  |  | ⚫ |  |  | ⚫ |  | ⚫ |  |  |  | 7 |
| Ruck  2019 | ⚫ |  |  |  | ⚫ |  |  |  | ⚫ |  |  |  | ⚫ | ⚫ |  |  | ⚫ |  |  | ⚫ |  |  | ⚫ |  |  | 7 |
| Kim  2019 |  | ⚫ |  |  |  |  |  | ⚫ |  |  |  |  | ⚫ | ⚫ |  |  | ⚫ |  |  |  | ⚫ | ⚫ |  |  |  | 5 |
| Frau  2019 |  | ⚫ |  |  |  |  |  |  | ⚫ |  |  | ⚫ |  | ⚫ |  |  | ⚫ |  |  |  | ⚫ |  | ⚫ |  |  | 6 |
| Alcalá  2019 | ⚫ |  |  |  | ⚫ |  |  |  | ⚫ |  |  | ⚫ |  | ⚫ |  |  | ⚫ |  |  |  | ⚫ |  | ⚫ |  |  | 7 |
| Wang  2018 | ⚫ |  |  |  | ⚫ |  |  |  | ⚫ |  |  | ⚫ |  | ⚫ |  |  | ⚫ |  |  |  | ⚫ |  |  |  | ⚫ | 6 |
| Prosperini  2018 | ⚫ |  |  |  |  |  |  |  | ⚫ |  |  | ⚫ |  | ⚫ |  |  | ⚫ |  |  | ⚫ |  | ⚫ |  |  |  | 7 |
| Pariani  2018 |  | ⚫ |  |  |  |  |  | ⚫ |  |  |  | ⚫ |  | ⚫ |  |  | ⚫ |  |  | ⚫ |  |  |  |  | ⚫ | 6 |
| Muller  2018 |  | ⚫ |  |  |  |  |  | ⚫ |  |  |  | ⚫ |  | ⚫ |  |  | ⚫ |  |  | ⚫ |  | ⚫ |  |  |  | 7 |
| Kocsik  2018 |  | ⚫ |  |  |  |  |  | ⚫ |  |  |  | ⚫ |  | ⚫ |  |  |  |  | ⚫ | ⚫ |  | ⚫ |  |  |  | 6 |
| Willis  2016 | ⚫ |  |  |  |  |  |  |  | ⚫ |  |  | ⚫ |  | ⚫ |  |  | ⚫ |  |  | ⚫ |  | ⚫ |  |  |  | 7 |
| Le Page  2015 |  | ⚫ |  |  |  |  |  |  | ⚫ |  |  | ⚫ |  | ⚫ |  |  | ⚫ |  |  | ⚫ |  | ⚫ |  |  |  | 7 |
| 1) Representativeness of the exposed cohort; 2) Selection of the non exposed cohort; 3) Ascertainment of exposure; 4) Demonstration that outcome of interest was not present at start study; 5) Comparability of cohorts on the basis of the design or analysis; 6) Assessment of outcome; 7) Was follow-up long enough for outcomes to occur; 8) Adequacy of follow up of cohorts. 1a) Truly representatively of the average in the community; 1b) somewhat representative of the average in the community; 1c) selected group of users, for example, nurses, volunteers; 1d) no description of the derivation of the cohort; 2a) drawn from the same community as the exposed cohort; 2b) drawn from a different source; 2c) no description of the derivation of the non exposed cohort; 3a) secure record (e.g. surgical records); 3b) structures interview; 3c) written self report; 3d) no description; 4a) yes; 4b) no; 5a) study controls for the most important factor; 5b) study control for any additional factor (This criteria could be modified to indicate specific control for a second important factor.); 6a) independent blind assessment; 6b) record linkage; 6c) self report; 6d) no description; 7a) yes (select an adequate follow up period for outcome of interest); 7b) no; 8a) complete follow up all subjects accounts for; 8b) subject lost to follow up unlikely to introduce bias-small number lost >85% follow up, or description provided of those lost; 8c) follow up rate <85% and no description of those lost; 8d) no statement. | | | | | | | | | | | | | | | | | | | | | | | | | | |
