# Supplementary material for: Risk of secondary autoimmune diseases with alemtuzumab treatment for multiple sclerosis: a systematic review and meta-analysis
Source: Front Immunol. 2024 Apr 16;15:1343971. doi: 10.3389/fimmu.2024.1343971 (PMC11058189; doi:10.3389/fimmu.2024.1343971)
Supplement: Supplementary file 5 [file Table_3.docx]

PubMed:

("Autoimmunity"[MeSH Terms] OR "Autoimmune Diseases"[MeSH Terms]) AND "Alemtuzumab"[MeSH Terms] AND "Multiple Sclerosis"[MeSH Terms]

OVID:

1.(secondary autoimmunity or Autoimmunity or Autoimmune Diseases).af.

2.Alemtuzumab.af.

3.Multiple Sclerosis.af.

4.1 and 2 and 3

EMBASE：

1.'multiple sclerosis'/exp AND 'alemtuzumab'/exp

2.'autoimmunity'/exp OR 'autoimmune disease'/exp

3.'secondary autoimmunity'

4.#2 OR #3

5.#1 AND #4

WOS:

1.(ALL=( secondary autoimmunity) OR ALL=(Autoimmunity) OR ALL=(Autoimmune Diseases)

2.ALL=(Alemtuzumab)

3.ALL=(Multiple Sclerosis)

4.#1 AND #2 AND #3
